# 6-Bromo-1-methyl-4-[2-(4-nitro­benzyl­idene)hydrazin-1-yl­idene]-2,2-dioxo-3,4-dihydro-1*H*-2λ^6^,1-benzothia­zine

**DOI:** 10.1107/S1600536812035374

**Published:** 2012-08-15

**Authors:** Muhammad Shafiq, William T. A. Harrison, Islam Ullah Khan

**Affiliations:** a Department of Chemistry, Government College University, Faisalabad 38000, Pakistan; b Department of Chemistry, University of Aberdeen, Mston Walk, Aberdeen AB24 3UE, Scotland; c Materials Chemistry Laboratory, Department of Chemistry, Government College University, Lahore, Pakistan

## Abstract

In the title compound, C_16_H_13_BrN_4_O_4_S, the dihedral angle between the aromatic rings is 4.1 (2)° and the C=N—N=C torsion angle is 175.5 (3)°. The nitro group is almost coplanar with the benzene ring to which it is attached [dihedral angle = 2.9 (7)°]. The thia­zine ring has an S-envelope conformation with the S atom displaced by 0.819 (3) Å from the mean plane of the other five atoms (r.m.s. deviation = 0.017 Å). In the crystal, C—H⋯O inter­actions link the mol­ecules and weak aromatic π–π stacking [centroid–centroid separation = 3.874 (2) Å] is also observed.

## Related literature

For the synthesis and biological activity of the title compound and related materials, see: Shafiq, Zia-ur-Rehman *et al.* (2011 ▶). For related structures, see: Shafiq, Khan *et al.* (2011 ▶); Shafiq, Harrison *et al.* (2012 ▶).
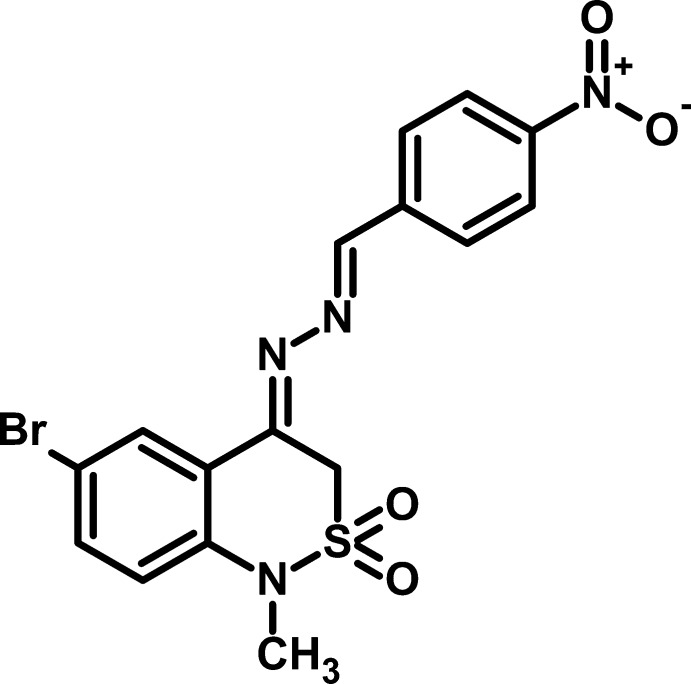


## Experimental

### 

#### Crystal data

C_16_H_13_BrN_4_O_4_S
*M* *_r_* = 437.27
Triclinic, 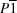

*a* = 8.2772 (4) Å
*b* = 9.0572 (4) Å
*c* = 12.6868 (6) Å
α = 87.132 (4)°
β = 70.976 (3)°
γ = 75.098 (2)°
*V* = 868.32 (7) Å^3^
*Z* = 2
Mo *K*α radiation
μ = 2.52 mm^−1^
*T* = 296 K
0.36 × 0.09 × 0.07 mm

#### Data collection

Bruker APEXII CCD diffractometer
Absorption correction: multi-scan (*SADABS*; Bruker, 2007 ▶) *T*_min_ = 0.464, *T*_max_ = 0.843
17474 measured reflections
4251 independent reflections
2484 reflections with *I* > 2σ(*I*)
*R*_int_ = 0.043

#### Refinement

*R*[*F* ^2^ > 2σ(*F* ^2^)] = 0.044
*wR*(*F* ^2^) = 0.106
*S* = 1.00
4251 reflections
236 parameters
H-atom parameters constrained
Δρ_max_ = 0.58 e Å^−3^
Δρ_min_ = −0.40 e Å^−3^

### 

Data collection: *APEX2* (Bruker, 2007 ▶); cell refinement: *SAINT* (Bruker, 2007 ▶); data reduction: *SAINT*; program(s) used to solve structure: *SHELXS97* (Sheldrick, 2008 ▶); program(s) used to refine structure: *SHELXL97* (Sheldrick, 2008 ▶); molecular graphics: *ORTEP-3* (Farrugia, 1997 ▶); software used to prepare material for publication: *SHELXL97*.

## Supplementary Material

Crystal structure: contains datablock(s) global, I. DOI: 10.1107/S1600536812035374/pv2578sup1.cif

Structure factors: contains datablock(s) I. DOI: 10.1107/S1600536812035374/pv2578Isup2.hkl

Supplementary material file. DOI: 10.1107/S1600536812035374/pv2578Isup3.cml

Additional supplementary materials:  crystallographic information; 3D view; checkCIF report

## Figures and Tables

**Table 1:** Hydrogen-bond geometry (Å, °)

*D*—H⋯*A*	*D*—H	H⋯*A*	*D*⋯*A*	*D*—H⋯*A*
C2—H2⋯O3^i^	0.93	2.56	3.325 (4)	139
C8—H8*B*⋯O2^ii^	0.97	2.44	3.310 (4)	150
C13—H13⋯O4^iii^	0.93	2.53	3.306 (5)	141
C15—H15⋯O2^iv^	0.93	2.53	3.426 (4)	162
C16—H16⋯O1^v^	0.93	2.45	3.218 (4)	139

Symmetry codes: (i) 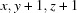; (ii) 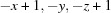; (iii) 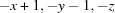; (iv) 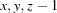; (v) 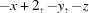.

**Fig. 1.**
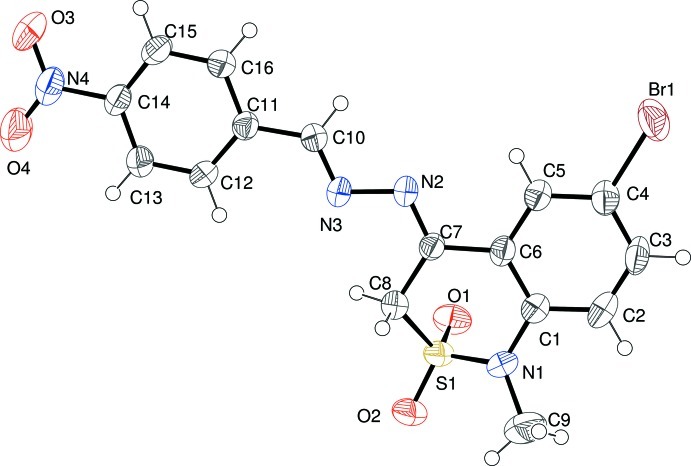
The molecular structure of the title compound with the atom numbering scheme. Displacement ellipsoids are drawn at the 50% probability level. H atoms are presented as small spheres of arbitrary radius.

**Fig. 2.**
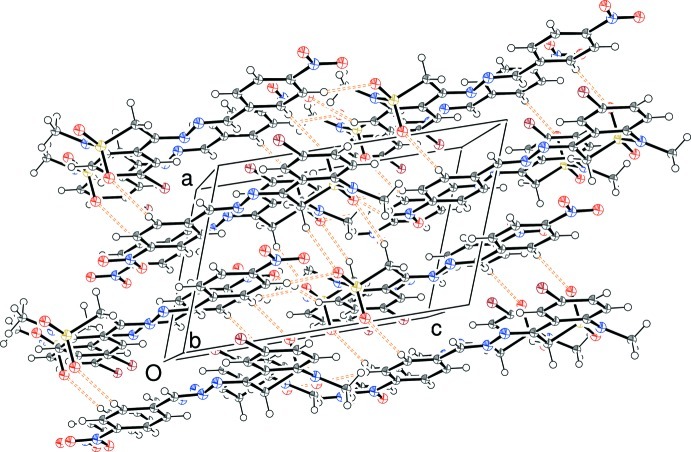
A view of the C—-H···O hydrogen bonds (orange double-dashed lines) in the crystal structure of the title compound.

## supplementary crystallographic information

### Comment

A number of benzothiazine derivatives have been found to be effective as drugs (Shafiq, Zia-ur-Rehman *et al.*, 2011). As a part of our ongoing studies in this area, we now describe the crystal structure of the title compound in this areticle.

The bond distances and angles in the title compound (Fig. 1) agree very well with the corresponding bond distances and angles reported in closely related compounds (Shafiq, Khan *et al.*, 2011; Shafiq, Harrison *et al.*, 2012). In the title molecule, the dihedral angle between the aromatic rings (C1–C6 and C11–C16) is 4.1 (2)° and the C7═N2—N3═C10 torsion angle is 175.5 (3)°. The nitro group is almost coplanar with benzene ring ((C11–C16) to which it is attached [dihedral angle = 2.9 (7)°]. The thiazine ring adopts an S-envelope conformation with S1 atom displaced by 0.819 (3) Å from the mean plane of the other five atoms (r.m.s. deviation = 0.017 Å).

The crystal packing (Fig. 2) is stabilized by weak intermolecular C—H···O hydrogen bonds (Table 1) linking the molecules to generate a three-dimensional network with all four O atoms acting as acceptors. Weak aromatic π-π stacking [centroid-centroid separation = 3.874 (2) Å] is also observed.

### Experimental

The compound was synthesized following the literature procedure (Shafiq, Zia-ur-Rehman *et al.*, 2011) and recrystalized from an ethylacetate solution under slow evaporation.

### Refinement

The H atoms were placed in calculated positions (C—H = 0.93–0.97 Å) and refined as riding. The methyl group was allowed to rotate, but not to tip, to best fit the electron density. The constraint *U*_iso_(H) = 1.2*U*_eq_(C) or 1.5*U*_eq_(methyl C) was applied. The (0 0 1) and (0 1 0) reflections were obstructed by the beamstop and were omitted from the refinement.

### Crystal data

**Table:**

C_16_H_13_BrN_4_O_4_S	*Z* = 2
*M**_r_* = 437.27	*F*(000) = 440
Triclinic, *P*1	*D*_x_ = 1.672 Mg m^−^^3^
Hall symbol: -P 1	Mo *K*α radiation, λ = 0.71073 Å
*a* = 8.2772 (4) Å	Cell parameters from 4062 reflections
*b* = 9.0572 (4) Å	θ = 2.3–22.1°
*c* = 12.6868 (6) Å	µ = 2.52 mm^−^^1^
α = 87.132 (4)°	*T* = 296 K
β = 70.976 (3)°	Needle, yellow
γ = 75.098 (2)°	0.36 × 0.09 × 0.07 mm
*V* = 868.32 (7) Å^3^	

### Data collection

**Table:**

Bruker APEXII CCD diffractometer	4251 independent reflections
Radiation source: fine-focus sealed tube	2484 reflections with *I* > 2σ(*I*)
Graphite monochromator	*R*_int_ = 0.043
ω scans	θ_max_ = 28.4°, θ_min_ = 2.7°
Absorption correction: multi-scan (*SADABS*; Bruker, 2007)	*h* = −11→11
*T*_min_ = 0.464, *T*_max_ = 0.843	*k* = −12→12
17474 measured reflections	*l* = −16→16

### Refinement

**Table:**

Refinement on *F*^2^	Primary atom site location: structure-invariant direct methods
Least-squares matrix: full	Secondary atom site location: difference Fourier map
*R*[*F*^2^ > 2σ(*F*^2^)] = 0.044	Hydrogen site location: inferred from neighbouring sites
*wR*(*F*^2^) = 0.106	H-atom parameters constrained
*S* = 1.00	*w* = 1/[σ^2^(*F*_o_^2^) + (0.0415*P*)^2^ + 0.5116*P*] where *P* = (*F*_o_^2^ + 2*F*_c_^2^)/3
4251 reflections	(Δ/σ)_max_ = 0.001
236 parameters	Δρ_max_ = 0.58 e Å^−^^3^
0 restraints	Δρ_min_ = −0.40 e Å^−^^3^

### Special details

**Table:**

Geometry. All e.s.d.'s (except the e.s.d. in the dihedral angle between two l.s. planes) are estimated using the full covariance matrix. The cell e.s.d.'s are taken into account individually in the estimation of e.s.d.'s in distances, angles and torsion angles; correlations between e.s.d.'s in cell parameters are only used when they are defined by crystal symmetry. An approximate (isotropic) treatment of cell e.s.d.'s is used for estimating e.s.d.'s involving l.s. planes.
Refinement. Refinement of *F*^2^ against ALL reflections. The weighted *R*-factor *wR* and goodness of fit *S* are based on *F*^2^, conventional *R*-factors *R* are based on *F*, with *F* set to zero for negative *F*^2^. The threshold expression of *F*^2^ > σ(*F*^2^) is used only for calculating *R*-factors(gt) *etc*. and is not relevant to the choice of reflections for refinement. *R*-factors based on *F*^2^ are statistically about twice as large as those based on *F*, and *R*- factors based on ALL data will be even larger.

### Fractional atomic coordinates and isotropic or equivalent isotropic displacement parameters (Å^2^)

**Table:**

	*x*	*y*	*z*	*U*_iso_*/*U*_eq_	
C1	0.8000 (4)	0.2966 (4)	0.3910 (3)	0.0429 (8)	
C2	0.8376 (5)	0.4239 (4)	0.4267 (3)	0.0591 (10)	
H2	0.8240	0.4365	0.5017	0.071*	
C3	0.8940 (5)	0.5298 (4)	0.3531 (3)	0.0516 (9)	
H3	0.9165	0.6146	0.3784	0.062*	
C4	0.9175 (4)	0.5116 (3)	0.2422 (3)	0.0443 (8)	
C5	0.8801 (4)	0.3880 (3)	0.2041 (3)	0.0385 (7)	
H5	0.8956	0.3769	0.1287	0.046*	
C6	0.8192 (4)	0.2795 (3)	0.2783 (2)	0.0371 (7)	
C7	0.7760 (4)	0.1511 (3)	0.2351 (2)	0.0336 (7)	
C8	0.7034 (4)	0.0381 (4)	0.3145 (2)	0.0438 (8)	
H8A	0.7315	−0.0590	0.2750	0.053*	
H8B	0.5757	0.0744	0.3446	0.053*	
C9	0.6400 (6)	0.2323 (5)	0.5848 (3)	0.0799 (13)	
H9A	0.7166	0.2499	0.6229	0.120*	
H9B	0.5874	0.1518	0.6195	0.120*	
H9C	0.5488	0.3242	0.5886	0.120*	
C10	0.7637 (4)	0.0248 (3)	−0.0055 (3)	0.0390 (7)	
H10	0.8022	0.1039	−0.0472	0.047*	
C11	0.7208 (4)	−0.0903 (3)	−0.0607 (2)	0.0362 (7)	
C12	0.6593 (4)	−0.2102 (4)	−0.0032 (2)	0.0413 (7)	
H12	0.6451	−0.2187	0.0724	0.050*	
C13	0.6194 (4)	−0.3163 (4)	−0.0580 (3)	0.0454 (8)	
H13	0.5775	−0.3964	−0.0199	0.054*	
C14	0.6428 (4)	−0.3016 (4)	−0.1703 (3)	0.0404 (7)	
C15	0.7034 (4)	−0.1855 (4)	−0.2294 (3)	0.0431 (8)	
H15	0.7184	−0.1782	−0.3052	0.052*	
C16	0.7415 (4)	−0.0794 (4)	−0.1731 (2)	0.0414 (8)	
H16	0.7820	0.0010	−0.2116	0.050*	
S1	0.79259 (11)	0.01247 (9)	0.42342 (6)	0.0432 (2)	
O1	0.9788 (3)	−0.0430 (3)	0.37576 (19)	0.0558 (6)	
O2	0.7010 (3)	−0.0721 (3)	0.50933 (18)	0.0569 (6)	
O3	0.6135 (4)	−0.3959 (3)	−0.3268 (2)	0.0759 (8)	
O4	0.5483 (5)	−0.5195 (3)	−0.1767 (3)	0.0898 (10)	
N1	0.7408 (4)	0.1889 (3)	0.4696 (2)	0.0498 (7)	
N2	0.7990 (3)	0.1447 (3)	0.1299 (2)	0.0404 (6)	
N3	0.7509 (3)	0.0216 (3)	0.0964 (2)	0.0396 (6)	
N4	0.5998 (4)	−0.4145 (4)	−0.2289 (3)	0.0559 (8)	
Br1	1.00058 (6)	0.65482 (4)	0.13996 (3)	0.06700 (17)	

### Atomic displacement parameters (Å^2^)

**Table:**

	*U*^11^	*U*^22^	*U*^33^	*U*^12^	*U*^13^	*U*^23^
C1	0.048 (2)	0.0466 (19)	0.0369 (18)	−0.0160 (15)	−0.0144 (15)	−0.0020 (15)
C2	0.076 (3)	0.063 (2)	0.046 (2)	−0.022 (2)	−0.0239 (19)	−0.0153 (18)
C3	0.069 (2)	0.0375 (18)	0.058 (2)	−0.0180 (17)	−0.0296 (18)	−0.0092 (16)
C4	0.051 (2)	0.0363 (17)	0.052 (2)	−0.0126 (15)	−0.0240 (16)	0.0005 (15)
C5	0.0415 (19)	0.0375 (16)	0.0399 (18)	−0.0096 (14)	−0.0175 (14)	−0.0018 (14)
C6	0.0370 (18)	0.0389 (17)	0.0387 (17)	−0.0117 (14)	−0.0143 (13)	−0.0046 (13)
C7	0.0338 (17)	0.0365 (16)	0.0320 (17)	−0.0101 (13)	−0.0115 (13)	−0.0018 (13)
C8	0.050 (2)	0.0496 (19)	0.0379 (18)	−0.0230 (16)	−0.0139 (15)	0.0011 (15)
C9	0.094 (3)	0.081 (3)	0.046 (2)	−0.023 (2)	0.006 (2)	−0.013 (2)
C10	0.0408 (19)	0.0436 (17)	0.0361 (18)	−0.0185 (14)	−0.0109 (14)	0.0002 (14)
C11	0.0338 (17)	0.0423 (17)	0.0352 (17)	−0.0125 (13)	−0.0121 (13)	−0.0018 (13)
C12	0.049 (2)	0.0495 (19)	0.0304 (17)	−0.0177 (15)	−0.0161 (14)	0.0016 (14)
C13	0.056 (2)	0.0424 (18)	0.046 (2)	−0.0217 (16)	−0.0201 (16)	0.0032 (15)
C14	0.0412 (19)	0.0451 (18)	0.0381 (18)	−0.0107 (15)	−0.0158 (14)	−0.0079 (14)
C15	0.0395 (19)	0.060 (2)	0.0315 (17)	−0.0118 (16)	−0.0128 (14)	−0.0055 (15)
C16	0.0431 (19)	0.0498 (19)	0.0353 (18)	−0.0197 (15)	−0.0123 (14)	0.0032 (14)
S1	0.0471 (5)	0.0504 (5)	0.0333 (4)	−0.0180 (4)	−0.0110 (3)	0.0065 (4)
O1	0.0443 (15)	0.0642 (15)	0.0522 (14)	−0.0085 (11)	−0.0126 (11)	0.0132 (12)
O2	0.0661 (17)	0.0680 (16)	0.0400 (13)	−0.0306 (13)	−0.0132 (11)	0.0155 (11)
O3	0.100 (2)	0.091 (2)	0.0488 (17)	−0.0368 (17)	−0.0284 (15)	−0.0191 (15)
O4	0.143 (3)	0.076 (2)	0.085 (2)	−0.063 (2)	−0.055 (2)	0.0059 (17)
N1	0.064 (2)	0.0584 (18)	0.0280 (14)	−0.0241 (15)	−0.0083 (12)	−0.0028 (12)
N2	0.0479 (17)	0.0391 (14)	0.0405 (16)	−0.0188 (12)	−0.0163 (12)	−0.0023 (12)
N3	0.0482 (16)	0.0375 (14)	0.0382 (16)	−0.0167 (12)	−0.0157 (12)	−0.0036 (11)
N4	0.058 (2)	0.060 (2)	0.057 (2)	−0.0167 (16)	−0.0241 (15)	−0.0144 (16)
Br1	0.0932 (4)	0.0392 (2)	0.0830 (3)	−0.0297 (2)	−0.0394 (2)	0.01409 (18)

### Geometric parameters (Å, º)

**Table:**

C1—C6	1.398 (4)	C10—N3	1.262 (4)
C1—C2	1.400 (4)	C10—C11	1.459 (4)
C1—N1	1.420 (4)	C10—H10	0.9300
C2—C3	1.367 (5)	C11—C16	1.380 (4)
C2—H2	0.9300	C11—C12	1.391 (4)
C3—C4	1.368 (4)	C12—C13	1.376 (4)
C3—H3	0.9300	C12—H12	0.9300
C4—C5	1.382 (4)	C13—C14	1.379 (4)
C4—Br1	1.884 (3)	C13—H13	0.9300
C5—C6	1.400 (4)	C14—C15	1.366 (4)
C5—H5	0.9300	C14—N4	1.470 (4)
C6—C7	1.478 (4)	C15—C16	1.381 (4)
C7—N2	1.288 (4)	C15—H15	0.9300
C7—C8	1.497 (4)	C16—H16	0.9300
C8—S1	1.748 (3)	S1—O1	1.420 (2)
C8—H8A	0.9700	S1—O2	1.423 (2)
C8—H8B	0.9700	S1—N1	1.631 (3)
C9—N1	1.443 (4)	O3—N4	1.217 (4)
C9—H9A	0.9600	O4—N4	1.219 (4)
C9—H9B	0.9600	N2—N3	1.402 (3)
C9—H9C	0.9600		
			
C6—C1—C2	119.1 (3)	N3—C10—H10	118.6
C6—C1—N1	121.3 (3)	C11—C10—H10	118.6
C2—C1—N1	119.7 (3)	C16—C11—C12	119.2 (3)
C3—C2—C1	121.0 (3)	C16—C11—C10	119.0 (3)
C3—C2—H2	119.5	C12—C11—C10	121.8 (3)
C1—C2—H2	119.5	C13—C12—C11	120.2 (3)
C2—C3—C4	120.2 (3)	C13—C12—H12	119.9
C2—C3—H3	119.9	C11—C12—H12	119.9
C4—C3—H3	119.9	C12—C13—C14	118.7 (3)
C3—C4—C5	120.3 (3)	C12—C13—H13	120.6
C3—C4—Br1	120.3 (2)	C14—C13—H13	120.6
C5—C4—Br1	119.3 (2)	C15—C14—C13	122.6 (3)
C4—C5—C6	120.4 (3)	C15—C14—N4	118.6 (3)
C4—C5—H5	119.8	C13—C14—N4	118.8 (3)
C6—C5—H5	119.8	C14—C15—C16	117.9 (3)
C1—C6—C5	118.9 (3)	C14—C15—H15	121.0
C1—C6—C7	122.2 (3)	C16—C15—H15	121.0
C5—C6—C7	118.9 (3)	C11—C16—C15	121.3 (3)
N2—C7—C6	117.6 (3)	C11—C16—H16	119.3
N2—C7—C8	123.0 (2)	C15—C16—H16	119.3
C6—C7—C8	119.4 (2)	O1—S1—O2	118.22 (15)
C7—C8—S1	110.5 (2)	O1—S1—N1	110.53 (15)
C7—C8—H8A	109.6	O2—S1—N1	108.09 (14)
S1—C8—H8A	109.6	O1—S1—C8	107.65 (15)
C7—C8—H8B	109.6	O2—S1—C8	110.70 (14)
S1—C8—H8B	109.6	N1—S1—C8	100.17 (15)
H8A—C8—H8B	108.1	C1—N1—C9	122.1 (3)
N1—C9—H9A	109.5	C1—N1—S1	116.7 (2)
N1—C9—H9B	109.5	C9—N1—S1	121.3 (2)
H9A—C9—H9B	109.5	C7—N2—N3	113.8 (2)
N1—C9—H9C	109.5	C10—N3—N2	112.1 (2)
H9A—C9—H9C	109.5	O3—N4—O4	123.4 (3)
H9B—C9—H9C	109.5	O3—N4—C14	117.9 (3)
N3—C10—C11	122.9 (3)	O4—N4—C14	118.7 (3)
			
C6—C1—C2—C3	−0.6 (5)	C13—C14—C15—C16	−0.2 (5)
N1—C1—C2—C3	−179.7 (3)	N4—C14—C15—C16	179.3 (3)
C1—C2—C3—C4	−1.1 (6)	C12—C11—C16—C15	−0.3 (5)
C2—C3—C4—C5	1.7 (5)	C10—C11—C16—C15	179.8 (3)
C2—C3—C4—Br1	−178.5 (3)	C14—C15—C16—C11	0.5 (5)
C3—C4—C5—C6	−0.5 (5)	C7—C8—S1—O1	−60.1 (3)
Br1—C4—C5—C6	179.6 (2)	C7—C8—S1—O2	169.3 (2)
C2—C1—C6—C5	1.7 (5)	C7—C8—S1—N1	55.4 (2)
N1—C1—C6—C5	−179.2 (3)	C6—C1—N1—C9	−149.0 (4)
C2—C1—C6—C7	−177.8 (3)	C2—C1—N1—C9	30.0 (5)
N1—C1—C6—C7	1.2 (5)	C6—C1—N1—S1	31.0 (4)
C4—C5—C6—C1	−1.2 (5)	C2—C1—N1—S1	−149.9 (3)
C4—C5—C6—C7	178.4 (3)	O1—S1—N1—C1	57.5 (3)
C1—C6—C7—N2	−179.6 (3)	O2—S1—N1—C1	−171.7 (2)
C5—C6—C7—N2	0.8 (4)	C8—S1—N1—C1	−55.9 (3)
C1—C6—C7—C8	2.2 (4)	O1—S1—N1—C9	−122.5 (3)
C5—C6—C7—C8	−177.4 (3)	O2—S1—N1—C9	8.3 (4)
N2—C7—C8—S1	148.6 (3)	C8—S1—N1—C9	124.2 (3)
C6—C7—C8—S1	−33.3 (4)	C6—C7—N2—N3	−178.5 (3)
N3—C10—C11—C16	−178.9 (3)	C8—C7—N2—N3	−0.4 (4)
N3—C10—C11—C12	1.2 (5)	C11—C10—N3—N2	−179.8 (3)
C16—C11—C12—C13	−0.1 (5)	C7—N2—N3—C10	175.5 (3)
C10—C11—C12—C13	179.7 (3)	C15—C14—N4—O3	−2.8 (5)
C11—C12—C13—C14	0.4 (5)	C13—C14—N4—O3	176.7 (3)
C12—C13—C14—C15	−0.3 (5)	C15—C14—N4—O4	178.9 (3)
C12—C13—C14—N4	−179.8 (3)	C13—C14—N4—O4	−1.6 (5)

### Hydrogen-bond geometry (Å, º)

**Table:**

*D*—H···*A*	*D*—H	H···*A*	*D*···*A*	*D*—H···*A*
C2—H2···O3^i^	0.93	2.56	3.325 (4)	139
C8—H8*B*···O2^ii^	0.97	2.44	3.310 (4)	150
C13—H13···O4^iii^	0.93	2.53	3.306 (5)	141
C15—H15···O2^iv^	0.93	2.53	3.426 (4)	162
C16—H16···O1^v^	0.93	2.45	3.218 (4)	139

Symmetry codes: (i) *x*, *y*+1, *z*+1; (ii) −*x*+1, −*y*, −*z*+1; (iii) −*x*+1, −*y*−1, −*z*; (iv) *x*, *y*, *z*−1; (v) −*x*+2, −*y*, −*z*.
